# High Pressure Restrains the Photo‐Induced Polyhedra Distortion in 0D Antimony‐Based Metal Halide

**DOI:** 10.1002/advs.202502189

**Published:** 2025-05-23

**Authors:** Jiaxiang Wang, Lingrui Wang, Weiqi Yuan, Yifang Yuan, Fei Wang, Kai Wang, Haizhong Guo

**Affiliations:** ^1^ Key Laboratory of Materials Physics Ministry of Education School of Physics Zhengzhou University Zhengzhou 450001 P. R. China; ^2^ Shandong Key Laboratory of Optical Communication Science and Technology School of Physics Science and Information Technology Liaocheng University Liaocheng 252059 P. R. China; ^3^ Institute of Quantum Materials and Physics Henan Academy of Sciences Zhengzhou 450046 P. R. China

**Keywords:** 0D, excitation‐dependent emission, high pressure, photo‐induced polyhedra distortion, metal halide

## Abstract

Recently, dual emission phenomena based on excitation‐dependent emission are increasingly observed in 0D antimony‐based metal halides. However, the origins of these two emissions remain debated. Herein, high pressure is employed to investigate the structural‐optical relationship of the typical 0D antimony‐based metal halides (TTA)_2_SbCl_5_ (TTA = tetraethylammonium) and uncover mechanisms behind its emission. As pressure increases, the dual emission of (TTA)_2_SbCl_5_ under 303 nm excitation, characterized by high energy and low energy emission peaks, transitions to a single low energy emission peak at ≈5.7 GPa. Meanwhile, a single broadband emission peak excited by 355 nm maintains a distinct low energy emission peak throughout the compression process, exhibiting an excitation‐dependent emission color response. Combining experiment results with density functional theory calculations, the observed phenomenon is attributed to pressure restrain of photo‐induced distortion in the [SbCl_5_]^2^⁻ polyhedra in (TTA)_2_SbCl_5_. These finding reveals the relationship between the local structure and excitation‐dependent emission, providing new perspectives and insights into the modulation of Sb‐based metal halides.

## Graphical abstract

0D antimony‐based metal halide (TTA)_2_SbCl_5_, exhibits dual emission at 303 nm excitation and a single emission peak at 355 nm excitation. From a high‐pressure perspective and in conjunction with density functional theory calculations, it is shown that the excitation‐dependent emission phenomenon in this is associated with photo‐induced polyhedra distortion, and that the pressure can effectively modulate such changes.

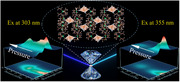

## 1 Introduction

In recent years, metal halides have garnered intensive attention for their excellent emissions properties. Generally, these emission properties are closely linked to their structural dimension. Previous studies have revealed that reducing dimensionality is a unique strategy to enhance the quantum confinement effect and boost the emission efficiency of metal halides from the molecular level.^[^ ^1^ ^]^ From this perspective, low‐dimensional, especially 0D metal halides, have exhibited great potential as luminescent materials with large Stokes‐shifted bright broadband emission due to self‐trapped excitons (STEs).^[^ ^2^ ^]^ In 0D metal halides, inorganic polyhedra are completely separated from each other and surrounded by large organic molecules or cations.^[^ ^3^ ^]^ These surrounding organic parts serve as a dielectric shielding layer, leading to weak electronic interactions between the isolated inorganic parts, thus making the inorganic parts act as isolated luminescent centers. Such isolated luminescent centers are characterized by large exciton binding energy, strong electron‐phonon coupling, and exhibit excitation‐dependent emission properties.^[^ ^4^ ^]^ Recently, researchers have found that Sb^3+^‐based 0D metal halides are receiving an increasing interest due to their special energy level distribution and diverse optical properties. Generally, these types of metal halides exhibit dual emission band (singlet and triplet STEs) or single emission bands (triplet STEs).^[^ ^5^ ^]^ However, it is still debated whether the broadband emission originates from specific ions or excited state defects that give rise to STEs.^[^ ^6^ ^]^ To date, there is still no effective methodology for modulating dual and single emission bands. Hence, there is an urgent need to gain deeper insight into the luminescence mechanisms and to achieve further optimization of Sb^3+^‐based metal halides for future applications.

High pressure is utilized as a clean means to continuously alter the crystal structure and properties without introducing component gradients, offering new perspectives for exploring materials with novel or enhanced properties.^[^ ^7^ ^]^ To date, extensive investigations have been conducted on metal halides, revealing various changes such as pressure‐induced emission (PIE), pressure‐induced emission enhancement (PIEE), and pressure‐induced stable emission, among others.^[^ ^8^ ^]^ For Sb^3+^‐based metal halides, their emission properties under high pressure are still in early stages of investigation. It has been demonstrated the Sb^3+^ exhibits colorful emission based on its coordination environment or the symmetry of the lattice.^[^ ^9^ ^]^ Herein, we choose the typical (TTA)_2_SbCl_5_ (TTA = tetraethylammonium) as a suitable platform to systematically and deeply understand the mechanism of broadband emission. Additionally, its unique excitation‐dependent emission properties offer perspectives for elucidating the relationship between dual and single emission bands.^[^ ^10^ ^]^

In this work, we analyze the excitation‐dependent broadband emission properties of the 0D antimony‐based metal halide (TTA)_2_SbCl_5_, revealing the relationship between the inorganic polyhedral structure and excitation‐dependent emission from a high pressure perspective, complemented by density functional theory (DFT) calculations. Under ambient conditions, excitation at 355 nm in (TTA)_2_SbCl_5_ results in broadband spectra featuring only low energy emission peaks, which decrease in intensity with increasing pressure. In contrast, excitation at 303 nm produces spectra with both high energy (Peak 2) and low energy (Peak 1) emission peaks. As pressure increases, the dual broadband emission transforms in to a single low energy peak (Peak 1) as the Peak 2 diminish. This behavior is attributed to pressure‐induced suppression of photogenic polyhedral distortions in inorganic polyhedra [SbCl₅]^2^⁻, as evidenced by simulations of excited state configurations under varying pressures. This work offers new insights into the excitation‐dependent emission properties in 0D Sb‐based metal halides and provides a deeper understanding of how to modulate their emission properties.

**Figure 1:**
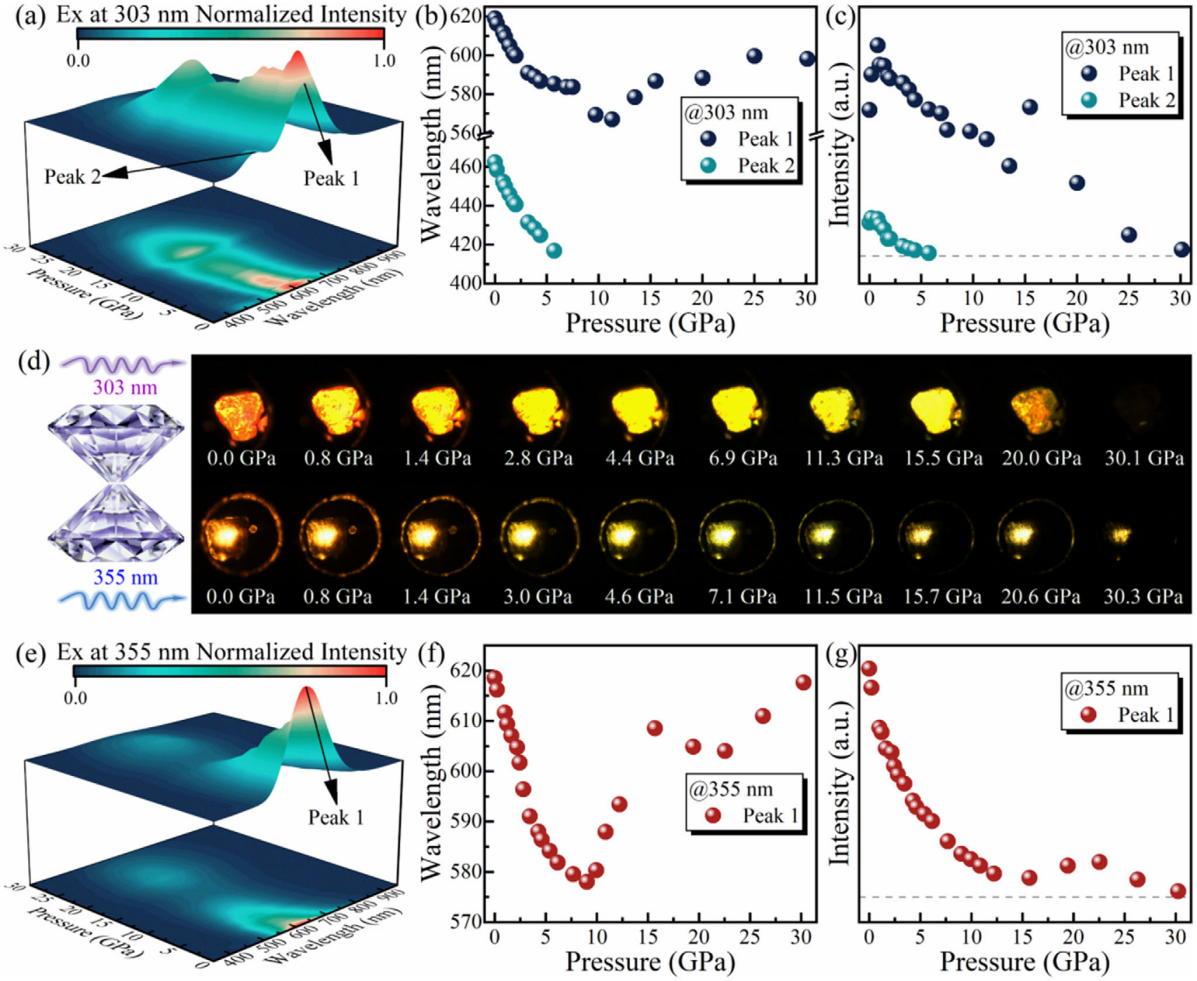
Pressure‐dependent PL spectra, along with the corresponding emission peak and emission intensity of (TTA)_2_SbCl_5_ under 303 nm (a–c) and 355 (e–g) nm excitation. Peak 1 represents the low energy emission peak, while Peak 2 represents the high energy emission peak. d) Schematic diagram of the diamond anvil cell and optical micrographs of (TTA)_2_SbCl_5_ under high pressure with excitation at 303 nm (top) and 355 nm (bottom), respectively.

As an excitation‐dependent emission metal halide, the pressure effect on the photoluminescence (PL) of (TTA)_2_SbCl_5_ is of particular interest to us in exploring its optical modulation mechanism. Initially, the excitation‐dependent emission properties of (TTA)_2_SbCl_5_ were analyzed using 3D excitation‐emission matrix PL spectroscopy under ambient conditions, as illustrated in Figure S1 (Supporting Information). We observed that the variation in excitation wavelengths leads to a shift in the position of the emission peak center, suggesting the presence of different emission mechanisms within this sample. To uncover the intrinsic effects of the above phenomenon, in situ high pressure PL experiments were subsequently conducted on (TTA)_2_SbCl_5_ crystal under high energy excitation at 303 nm and low energy excitation at 355 nm, respectively. To be specific, upon excitation at 303 nm, (TTA)_2_SbCl_5_ exhibited dual‐emission characteristics with a low energy emission peak (Peak 1, centered at 619 nm) and a high energy emission peak (Peak 2, centered at 462 nm) under ambient conditions (**Figure** **1**a). The photoluminescence excitation (PLE) spectra of Peak 1 and Peak 2 emissions, shown in Figure S2 (Supporting Information), visually illustrate the origins of their distinct emission characteristics. As pressure increased, Peak 1 displayed a blue shift to ≈11.3 GPa, after which it gradually red shifted (Figure 1b). Its intensity initially increased, then decreased, and eventually vanished at 30.1 GPa. In contrast, Peak 2 continued to exhibit a blue shift and its intensity gradually decreases, with the peak disappearing beyond 5.7 GPa (Figure 1c). However, under 355 nm excitation, (TTA)_2_SbCl_5_ displays a single emission center at 620 nm, which we also designate as Peak 1 (Figure 1e). This Peak 1 undergoes a gradual blue shift with a weaking of peak intensity up to 9.0 GPa, followed by a red shift in the pressure range of 9.0–15.7 GPa (Figure 1f,g). Beyond 15.7 GPa, there is a slight blue shift accompanied by an increase in intensity. An inflection point occurs at 22.6 GPa, after which Peak 1 exhibits a decreasing trend with a red shift until 30.3 GPa. Notably, the emission color of (TTA)_2_SbCl_5_ at high pressure shows a pronounced excitation‐dependent response (Figure 1d). To quantitatively describe the variation in emission color with pressure for both excitations, the Commission Internationale de l'Eclairage (CIE) chromaticity coordinates were recorded (Figure S3, Supporting Information). It is noteworthy that upon decompression to ambient conditions, the proportion of Peak 2 versus Peak1 produced under 303 nm excitation changed, and the centers of the emission peaks for both exhibited a slight red‐shift (Figure S4, Supporting Information). This suggests that some irreversible alterations in the crystal structure may have occurred.

**Figure 2:**
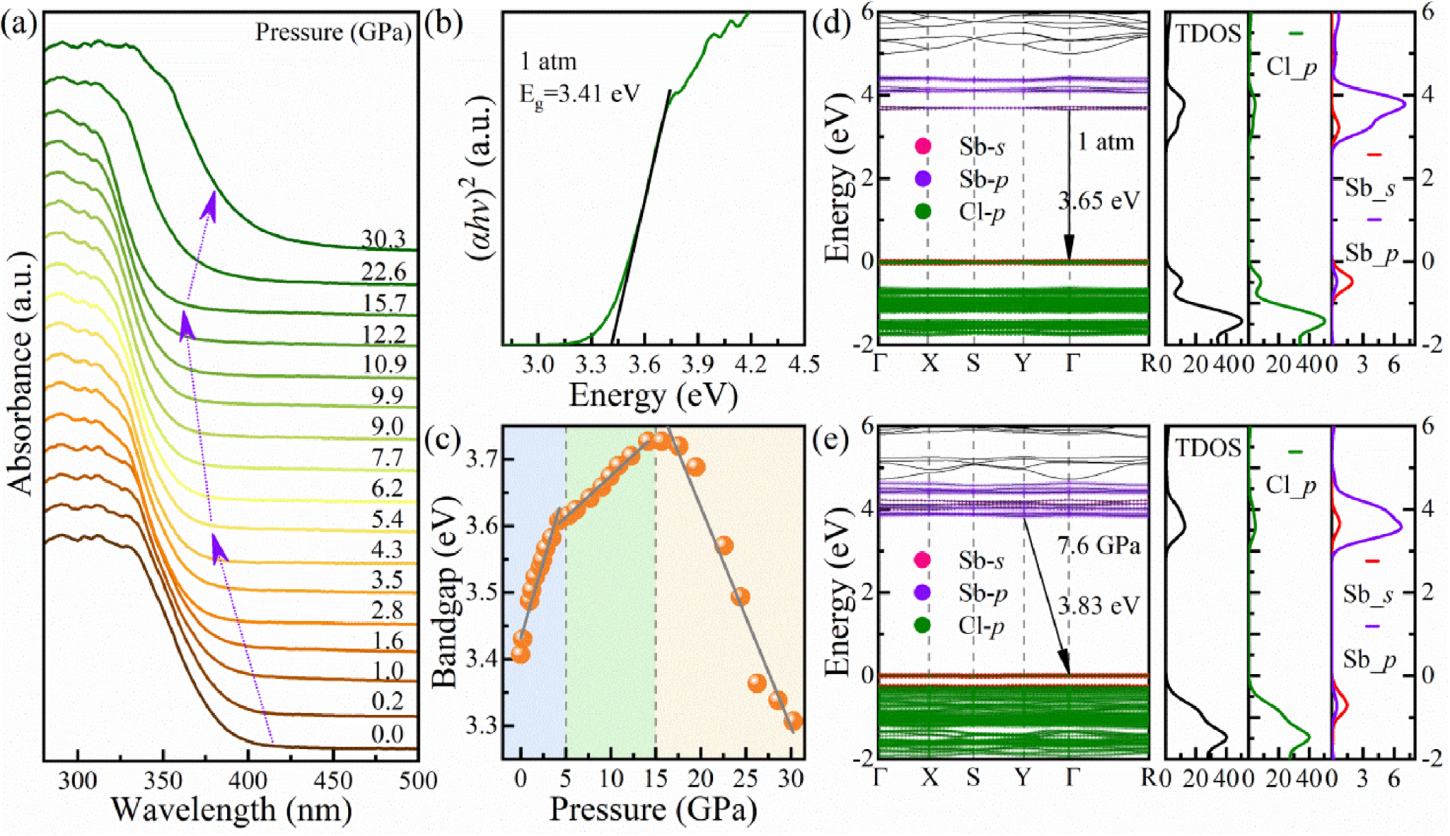
a) UV–vis absorption spectra of (TTA)_2_SbCl_5_ as a function of pressure. b) Tauc plot of bandgap using UV–vis spectrum at ambient conditions. c) Evolution of the bandgap of (TTA)_2_SbCl_5_ during compression. The calculated energy band structure d) and partial density of states (PDOS) (e) of (TTA)_2_SbCl_5_ at 1 atm and 7.6 GPa using density functional theory (DFT).

To further explore the optical properties of (TTA)_2_SbCl_5_, in situ high pressure UV–vis absorption spectroscopy experiments, combined with theoretical calculations, were conducted. As shown in **Figure** **2**a, the absorption edge of (TTA)_2_SbCl_5_ experiences undergoes a blue shift up to 15.0 GPa, followed by a red‐shift until 30.3 GPa. Their corresponding optical bandgap (*E*_g_) was estimated by fitting the Tauc plot.^[^ ^11^ ^]^ Figure 2b shows the bandgap of (TTA)_2_SbCl_5_ is ≈3.41 eV at ambient conditions. The evolution of the bandgap with pressure follows three distinct stages (Figure 2c): a rapid widening in the 0–5.4 GPa range (≈0.039 eV/GPa), a slower widening in the 5.4–15.7 GPa range (≈0.011 eV/GPa), a rapid narrowing in the 15.7–30.3 GPa range (≈0.029 eV/GPa). After releasing the pressure to ambient conditions, the bandgap slightly widens, corresponding to the PL anomaly observed before and after pressure release (Figure S5, Supporting Information). It also can be inferred that the disappearance of Peak 2 in the PL spectrum under 303 nm excitation around 5.4 GPa is closely related to this bandgap mutation. To further understand this pressure regulation of the metal halide bandgap, density functional theory (DFT) calculations were carried out. The calculated bandgaps of (TTA)_2_SbCl_5_ at 1 atm and 7.6 GPa are illustrated in Figure 2d,e, respectively. Under ambient conditions, (TTA)_2_SbCl_5_ exhibits a direct bandgap of 3.65 eV. The 5*s* and 5*p* orbitals of Sb, along with the 3*p* orbitals of Cl within the inorganic polyhedra [SbCl_5_]^2^⁻, contribute to both the conduction band minimum (CBM) and the valence band maximum (VBM). The calculated energy band structure suggests that (TTA)_2_SbCl_5_ exhibits limited electronic band dispersion and a larger effective mass of the carriers. We observed a broadened bandgap of 3.83 eV with the CBM elevated at 7.6 GPa, where the electronic band dispersion at the band edge increases and the carrier effective mass decreases. As a result, the overlap of the electron clouds of Sb and Cl may increase due to the distortion of the inorganic polyhedra [SbCl_5_]^2^⁻. This could be a potential reason for the excitation‐dependent emission phenomena as well as the disappearance of the Peak 2. Due to the minimal energy difference between the conduction band minimum and the next smallest conduction band, as well as the potential direct‐indirect bandgap transitions, a similar trend in the bandgap evolution with pressure was obtained using an indirect bandgap fit (Figure S6, Supporting Information).

**Figure 3:**
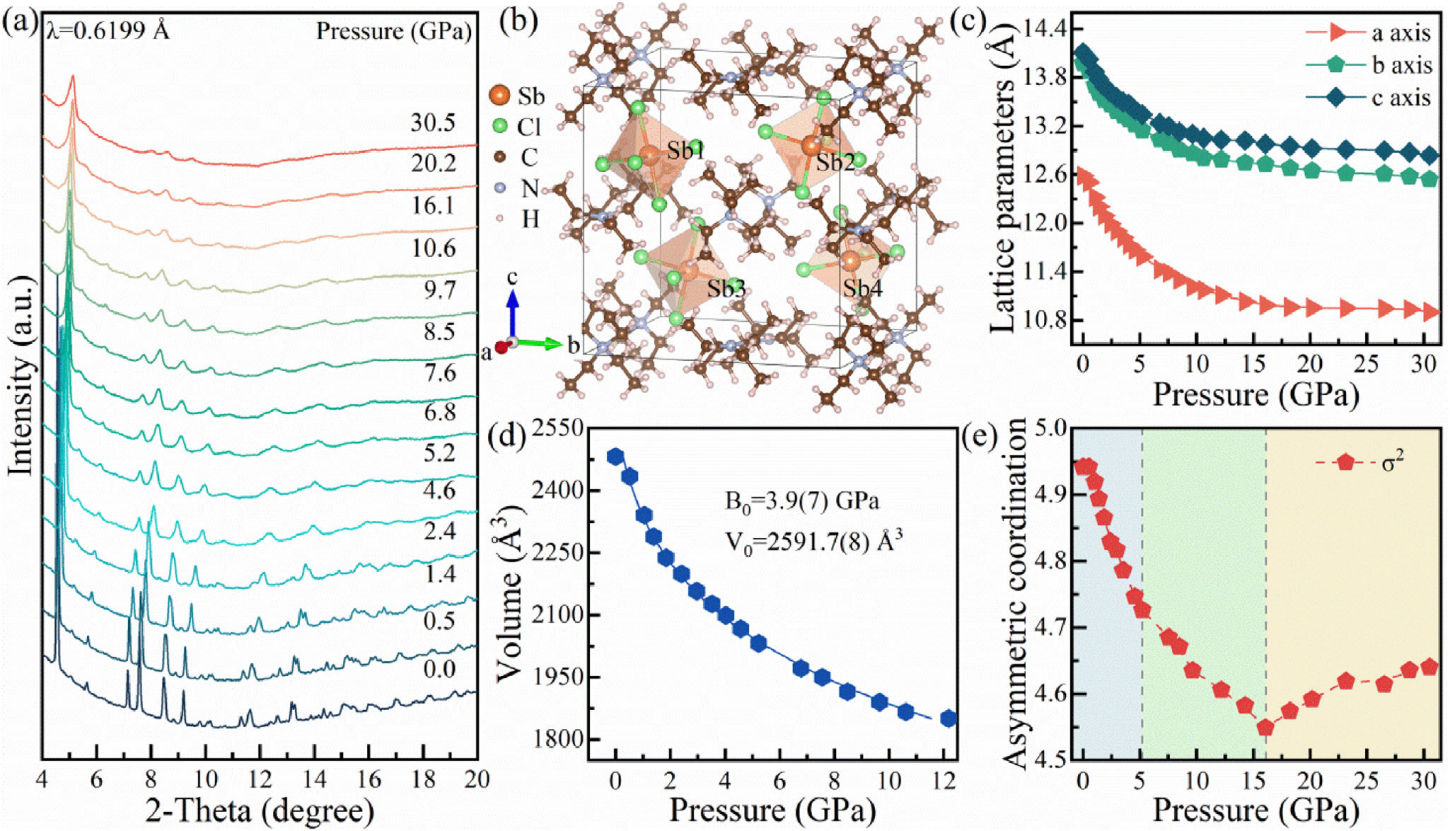
a) The representative angle‐dispersive x‐ray diffraction (ADXRD) patterns for (TTA)_2_SbCl_5_ as a function of pressure. b) The crystal structure of (TTA)_2_SbCl_5_. c) The lattice constant of (TTA)_2_SbCl_5_ with pressure. d) Evolution of the asymmetric coordination of [SbCl_5_]^2−^ inorganic polyhedra with pressure. e) The high pressure of lattice volume. The data is fitted by the third‐order Birch–Murnaghan equation fitting in different pressure ranges.

The changes in the energy band structure are closely linked to the crystal structure. Hence, to identify the reasons behind the relevant emission properties and the origin of the two emission peaks, in situ ADXRD experiments are conducted to analyze the structure optical property relationship. During compression to 30.5 GPa, all diffraction peaks shifted to higher angles, indicating lattice of (TTA)_2_SbCl_5_ continued to shrink (**Figure** **3**a). There are no new peaks are observed. To characterize the specific structure of (TTA)_2_SbCl_5_ under pressure, the Rietveld refinements were conducted based on high pressure ADXRD patterns (Figures S7–S9, Supporting Information). Under ambient conditions, (TTA)_2_SbCl_5_ possessed an orthorhombic structure with space group *Pnna*, and the lattice parameters were *a* = 12.58(5) Å, *b* = 13.97(6) Å, *c* = 14.10(7) Å, and *α* = *β* = *γ* = 90°. The crystal structure and coordinate axes of (TTA)_2_SbCl_5_ are illustrated in Figure 3b, where the [SbCl_5_]^2−^ polyhedra are fundamentally equivalent, and the neighboring polyhedra are numbered for better measure of the distance between different polyhedra. Accordingly, the STE emission of Sb‐based halides is found to be closely related to the distance of [SbCl_5_]^2−^ units and local unit distortion in the lattice. Concerned with the analysis of anomalous emission phenomena, the curves of lattice constants versus pressure were collected for (TTA)_2_SbCl_5_ before 30.5 GPa, as shown in Figure 3c. The shrinkage of the crystal lattice had a distinct anisotropy attributed to the differential compressibility of organic layers and the inorganic [SbCl_5_]^2−^ polyhedra. The distance between the closest [SbCl_5_]^2−^ polyhedra also exhibits no significant abrupt change with the pressure evolution. By fitting the unit‐cell volume to the Birch‐Murnaghan equation of state, a small bulk modulus (B_0_) of 3.9(7) GPa was determined for (TTA)_2_SbCl_5_, confirming the “soft” nature of the Sb‐based metal halides (Figure 3d).^[^ ^12^ ^]^ Interestingly, the distance between the nearest [SbCl_5_]^2−^ polyhedra does not show any significant abrupt change with increasing pressure (Figure S10, Supporting Information). Thus, it is likely that the variations within the polyhedra have a more significant effect on the Peak 2 than variations between the polyhedra. Based on this conjecture, the origin of the Peak 2 and the unique emission phenomena associated with pressure‐dependent excitation are analyzed by assessing the variation in [SbCl_5_]^2−^ polyhedral asymmetry with pressure. The magnitude of the degree of asymmetric coordination of the Sb^3+^ centers in [SbCl_5_]^2−^ polyhedra can be assessed using the following equation:^[^ ^10^, ^13^ ^]^

(1) $$\sigma^{2}=\frac{1}{7}\sum_{i=1}^{8}{\left(\alpha_{i}-90^{\circ }\right)}^{2}$$

where α_i_ is the Cl−Sb−Cl angles and σ^2^ is the degree of asymmetric coordination of the Sb^3+^ centers. Surprisingly, the asymmetric coordination of the individual [SbCl_5_]^2−^ polyhedra decreases rapidly up to 5.2 GPa, then declines more gradually between 5.2 GPa and 16.0 GPa, before beginning to increase slightly thereafter (Figure 3e). These variations closely resemble the changes in the optical bandgap with pressure. It can be concluded that the excitation‐dependent emission phenomenon, the disappearance of the Peak 2, and the pressure‐induced bandgap shifts are primarily governed by the degree of distortion within the isolated [SbCl_5_]^2−^ polyhedra. In addition, after releasing the pressure, the diffraction peak intensities recovered, but both shifted slightly to lower angles (Figure S11, Supporting Information). This effect of lattice expansion after pressure release suggests that an amorphization‐recrystallization process may have occurred during the compression and pressure release process.^[^ ^14^ ^]^ This may explain the change in the ratio of Peak 2 to Peak 1 under 303 nm excitation after the decompression.

**Figure 4:**
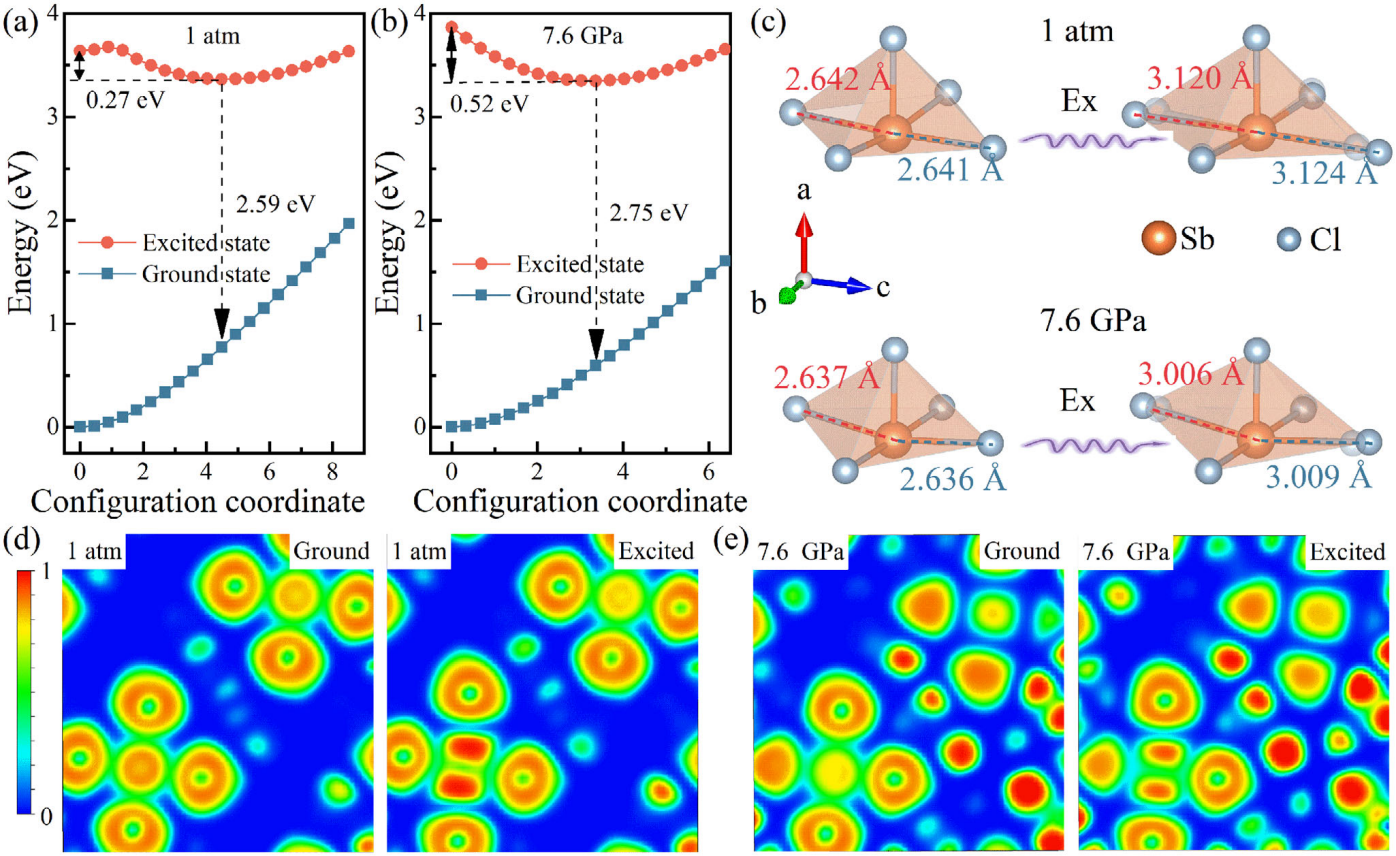
Configuration coordinate diagram of STE emission within the [SbCl_5_]^2−^ polyhedra at a) 1 atm and b) 7.6 GPa, respectively. c) Schematic of theoretically calculated inorganic [SbCl_5_]^2−^ polyhedra distortion under excitation 1 atm and 7.6 GPa. The electron localization function (ELF) of the ground and excited states at d) 1 atm and e) 7.6 GPa, respectively. The excited state structure of a single [SbCl_5_]^2−^ polyhedra is calculated due to the equivalence of [SbCl_5_]^2−^ polyhedra.

The analysis of the configuration coordinate results reveals a significant difference between the equilibrium positions of the excited and ground states under ambient conditions. Further theoretical analysis was carried out to simulate STE formation and structural distortions after excitation, as illustrated in **Figure** **4**. The [SbCl_5_]^2−^ polyhedra exhibit substantial structural distortions in the excited state, consistent with the observed large Stokes shift of broadband emission phenomenon at 1 atm (Figure 4a). Meanwhile, the configuration of the excited states suggests the presence of a metastable equilibrium configuration in the first excited state. This, along with the previously reported increase in the percentage of Peak 2 of (TTA)_2_SbCl_5_ at low temperatures, indicates that the high energy emission peaks are correlated with the metastable state's equilibrium configuration.^[^ ^10^ ^]^ Under high pressure, the excited state experiences a reduced shift in the configuration coordinate, as the pressure effectively suppresses the structural distortion of the excited state (Figure 4b).^[^ ^15^ ^]^ Consequently, the calculated PL emission energy increases from 2.59 eV at 1 atm to 2.75 eV at 7.6 GPa, which aligns with the observed blue shift of the emission peaks in the experimental results. Generally, conventional 3D metal halides have large polarons between neighboring inorganic octahedra.^[^ ^16^ ^]^ To verify the location of self‐trapped excitons and exclude the influence of the inorganic polyhedra between them, the positions of electron polarons and hole polarons were calculated at different pressures, as shown in Figure S12 (Supporting Information).^[^ ^17^ ^]^ Due to the dielectric shielding of the organic groups, both the electron polarons and hole polarons are located inside the [SbCl_5_]^2−^ inorganic polyhedra, and the Cl‐Sb‐Cl rows along the *c*‐axis direction are more compact. As a result, the bond lengths and bond angles of Cl‐Sb‐Cl along the *c*‐axis direction of the [SbCl_5_]^2−^ polyhedra change more drastically during excitation progress (Figure 4c). However, at high pressure, the [SbCl_5_]^2−^ polyhedra distortion is suppressed under excitation, resulting in the disappearance of the Peak 2. To visualize and analyze the structural symmetry, calculations of the electronic localization function (ELF) were performed for the ground and excited state structures at different pressures. Due to the presence of lone pair electrons in the coordination environment of Sb, the spatial electron distributions of the HOMO and the LUMO of [SbCl_5_]^2−^ were also calculated (Figure S13, Supporting Information). The lone pair of electrons behaves differently in the ground and excited states, and their changes induce changes in the degree of polyhedral distortion, so that the changes in the excited state depend mainly on the changes in the planes where the four Cl and one Sb are located, and the [100] direction was chosen for the observations (Figure 4d,e). Observations along the [010] direction are shown in Figure S14 (Supporting Information).

Based on the above discussion, a model is proposed to elucidate the excitation‐dependent emission phenomena from a high pressure perspective. When (TTA)_2_SbCl_5_ is excited with high energy light (303 nm), isolated luminescent centers [SbCl_5_]^2−^ undergo phototropic aberrations, leading to structural relaxation into the excited metastable state and the excited state of minimum energy. This behavior is reflected in the potential energy surface, corresponding to both the lowest excited state and the metastable energy site. In this case, (TTA)_2_SbCl_5_ exhibits both Peak 2 and Peak 1, with both displaying broadband emissions. Comparatively, with low energy excitation, the photo‐induced distortion in the polyhedra only relaxes to the lowest energy state of the excited configuration, as the distortion does not reach the excited metastable state configuration. This excitation‐dependent emission phenomenon reflects different relaxation processes of the excited state structure. Structurally, pressure suppresses this polyhedral distortion caused by excitation light, leading to a reduced potential barrier between the excited metastable state and the lowest energy state of the excited configuration. Consequently, the PL spectrum shifts from a broadband bimodal emission (Peak 1 and Peak 2) to a broadband single‐peak emission (Peak 1).

## 2 Conclusion

In conclusion, this study leverages high pressure to effectively investigate and analyze the structural distortions of the inorganic components in 0D Sb‐based metal halides, both in fundamental and excited states. By exploring the original of broadband emission and integrating theoretical calculations, a model is proposed to elucidate the excitation‐dependent emission properties and their relationship with the excited state structure. Under excitation at 303 nm, the observed dual emission spectra are attributed to metastable state on the excited state potential energy surface, polyhedral distortions induced by high energy excitation, and the storage of metastable configurations during relaxation. Conversely, low energy excitation produces spectra with only stable configurations corresponding to the lowest energy states on the excited state potential energy surface, due to smaller polyhedral distortions. This distortion is closely related to the different states of the lone pair of electrons in the ground and excited states. As pressure increase, the suppression of photo‐induced polyhedral distortion under high energy excitation leads to emission behaviors similar to those observed under low energy excitation. These findings not only elucidate the photophysical mechanisms behind the excitation‐dependent emission properties in (TTA)_2_SbCl_5_ but also offer insights into the optimal customization of broadband‐emission metal halides.

## Conflict of Interest

The authors declare no conflict of interest.

## Supporting information



Supporting Information

## Data Availability

The data that support the findings of this study are available in the supplementary material of this article.
