# Supplementary material for: High Pressure Restrains the Photo‐Induced Polyhedra Distortion in 0D Antimony‐Based Metal Halide
Source: Adv Sci (Weinh). 2025 May 23;12(30):e02189. doi: 10.1002/advs.202502189 (PMC12376567; doi:10.1002/advs.202502189)
Supplement: Supplementary file 1 — Supporting Information [file ADVS-12-e02189-s001.docx]

**Supporting Information**

**High Pressure Restrains the** **Photo-Induced Polyhedra Distortion in** **Zero-Dimensional Antimony-Based Metal Halide**

Jiaxiang Wang,^a^ Lingrui Wang,^a,^ * Weiqi Yuan,^a^ Yifang Yuan,^a^ Fei Wang,^a^ Kai Wang^b,^ * and Haizhong Guo^a, c,^ *

^a^ Key Laboratory of Materials Physics, Ministry of Education, School of Physics, Zhengzhou University, Zhengzhou 450001, P. R. China

^b^ Shandong Key Laboratory of Optical Communication Science and Technology, School of Physics Science and Information Technology, Liaocheng University, Liaocheng 252059, P. R. China

^c^ Institute of Quantum Materials and Physics, Henan Academy of Sciences, Zhengzhou 450046, P. R. China

Email: wanglr@zzu.edu.cn; kaiwang@lcu.edu.cn; hguo@zzu.edu.cn.

**Table of contents**

**Experiment details**2

**Computational details**5

**Supporting Figures**6

Figure S1. The photoluminescence and excitation maps of (TTA)_2_SbCl_5_ 6

Figure S2. The photoluminescent excitation of high energy emission (Peak 1) and low energy emission (Peak 2).7

Figure S3. Evolution of chromaticity coordinates with pressure under 303 nm and 355 nm excitation.8

Figure S4. The PL spectra before and after pressure treatment under 303 nm excitation 9

Figure S5. The direct bandgap before and after pressure treatment 10

Figure S6. The indirect bandgap Tauc plot of (TTA)_2_SbCl_5_ and bandgap evolution with pressure 11

Figure S7. Rietveld refinement results under partial pressure 12

Figure S8. Evolution of distances between different [SbCl_5_]^2-^ polyhedral Sb atoms with pressure 13

Figure S9. The ADXRD before and after pressure treatment 14

Figure S10. Wave function isosurface for electron and hole of (TTA)_2_SbCl_5_ in the excited state at 1 atm and 7.6 GPa.15

Figure S11. The electron localization function of (TTA)_2_SbCl_5_ along the [011] direction at 1 atm and 7.6 GPa.16

**References** 17

**Experiment details**

**Sample synthesis**

The (TTA)_2_SbCl_5_ crystals were synthesized according to the method that has been previously reported in the literature.^[1]^ Antimony trichloride (SbCl_3_, 0.5 mmol, 99.98%, Aladdin) and tetraethylammonium chloride ((C_2_H_5_)_4_NCl, 1.0 mmol, 98%, Aladdin) were added together to a solvent mixture of 1 ml of DMSO and 5 ml of DMF and heated at 60 ℃ to a clear solution. Bulk crystals were obtained by slowly diffusing ether into the above solution at room temperature using the antisolvent vapour diffusion method. After washing with dichloromethane, it was dried under vacuum for 12 hours.

**Generation of high pressure**

The high pressure environment was provided by symmetrical diamond anvil cells (DACs). Type II-a ultralow fluorescence diamonds with a culet diameter of 400 μm were used. The high pressure sample chamber was formed from a stainless-steel gasket with a pre-indented thickness of about 45 μm and a hole with a diameter of about 100 μm by laser-drilling the center part of it. One (TTA)_2_SbCl_5_ crystal (about 40×50 μm^2^) and a ruby ball (for pressure measurements) were loaded inside the sample chamber, and the pressures were monitored by the ruby fluorescence method. Mineral oil was used as the pressure transmitting medium.

***In situ* high pressure measurement**

*In situ* high pressure photoluminescence (PL) experiments were carried out by 303 nm and 355 nm excitation lasers, with additional components provided by Light & Microvision Industrial Technology Co., Ltd. Data were collected using the deuterium halogen light source and the Ocean Optics QE65000 fiber optic spectrometer. *In situ* PL micrographs of the samples were obtained using a camera (Canon Eos 5D mark II) equipped on a microscope (Ecilipse TI-U, Nikon).

UV-Vis absorption spectra were measured in the exciton absorption band region using a Deuterium-Halogen light source with wavelength range between 200 nm and 1700 nm. High-pressure absorption spectra of sample were also recorded with an optical fiber spectrometer (Ocean Optics, QE65000). The method of transmission absorption involves measuring the light intensity passing through the sample and a blank background. The difference between the two measurements is used to calculate the amount of light that was absorbed by the sample.

The *in situ* high pressure XRD experiments were performed at beamline 15U1 of Shanghai Synchrotron Radiation Facility (SSRF). The wavelength of the monochromatic X-ray beam is 0.6199 Å. Before the experiments, CeO_2_ was used as the standard sample for the geometry calibration. The diffraction patterns were integrated by the Dioptas software. All experiments were performed at room temperature.

**Data analyses**

The refinements of ADXRD patterns were performed using the Materials Studio program with Reflex module. All refinements were performed using four refinement cycles to satisfy the convergence criteria. First, the pattern was indexed by means of the peak picking option of the software package. Then, a profile-fitting procedure was applied to refine cell parameters and search space group. The refinements were performed to obtain the crystal structural parameters. The quality of the fitting between the experimental and calculated profile is assessed by the various *R* parameters like *R_p_* (profile factor) and *R_wp_* (weighted profile factor).

Fitting cell volume data to the Birch-Murnaghan equation of state:

where V_0_, V is the volume at ambient condition and high pressure, respectively; B_0_ is the bulk modulus at ambient condition, and B’ is the derivative of the bulk modulus with respect to pressure.

**Computational details**

The first-principles calculations based on density functional theory (DFT) were performed using the plane-wave pseudopotential as implemented in the Vienna Ab-initio Simulation Package (VASP).^[2]^ The electron-core interactions were described with the frozen-core projector-augmented wave pseudopotentials. The generalized gradient approximation formulated by Perdew, Burke, and Ernzerhof (PBE) as the exchange correlation functional with cutoff energies of 400 eV was chosen in all of our calculations.^[3]^ Appropriate Monkhorst-Pack k-meshes with grid spacing of 2π×0.03 Å^-1^ were chosen. The total energy convergence criteria of 1.0×10^-5^ eV and the force on each atom converge to 0.01 eV Å^-1^ in optimizing the geometric structure. The vdW interaction is considered by using the DFT-D2 method of Grimme.^[4]^ VASPKIT, an interface for VASP calculation, has been used in data processing. The self-trapped exciton emission of an exciton was obtained by taking the energy difference between the relaxed ground-state and excited-state structures, following the Franck-Condon principle. During the total energy calculation of an exciton, the occupation numbers of both the electron-occupied and hole-occupied eigenlevels were fixed using the delta self-consistent field (∆SCF) method.^[5]^ This approach enabled accurate predictions of the exciton emission properties in these low-dimensional halides perovskites.





**Figure S1**. The PL/PLE correlation maps of (TTA)_2_SbCl_5_ measured at ambient condition.





**Figure S2**. The PLE corresponding to Peak 1and Peak 2 at ambient conditions, respectively.


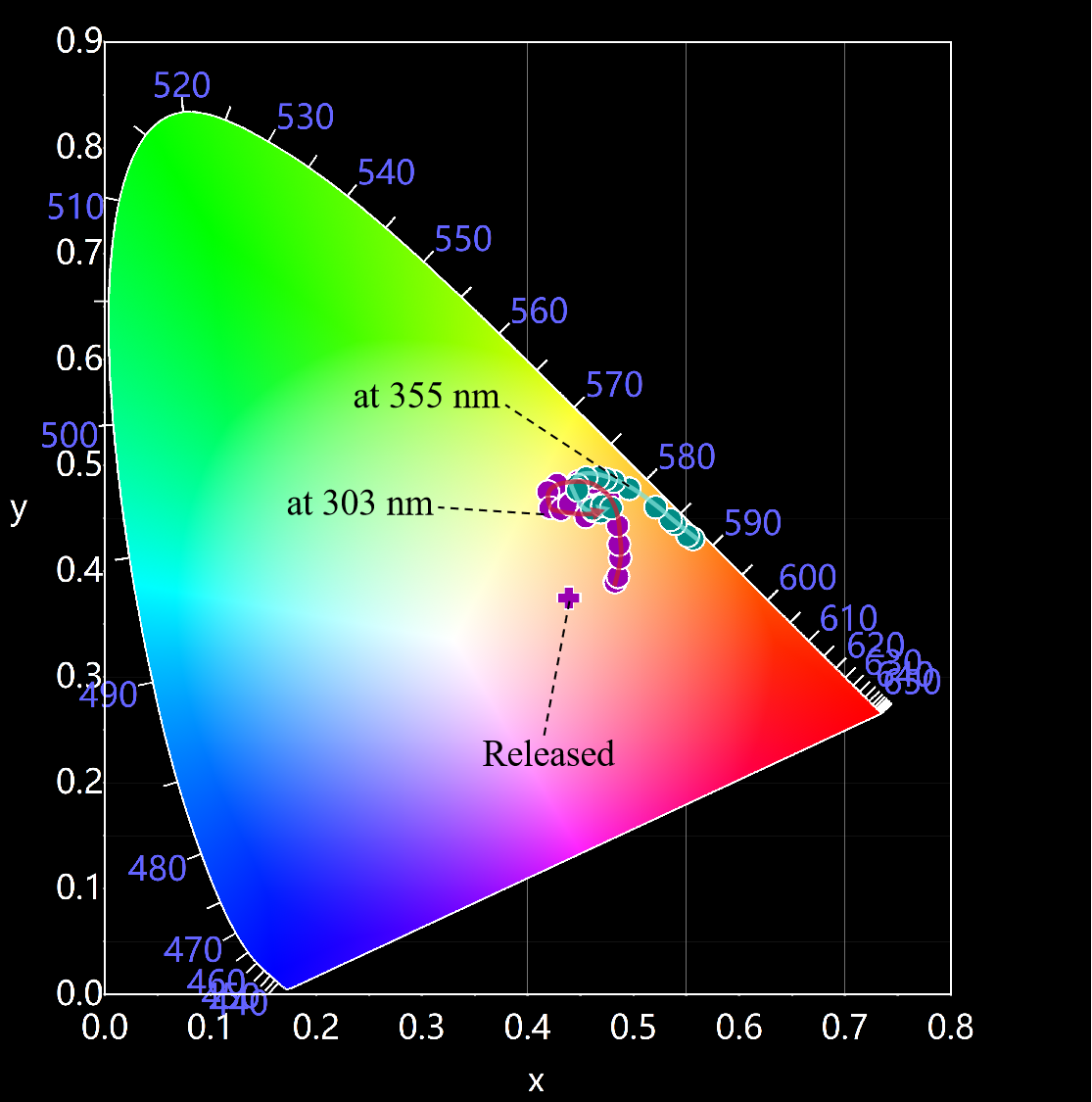


Figure S3. The variation of colour gamut with increasing pressure under 303nm and 355nm excitation.





Figure S4. The PL spectra of the initial state and after decompression under 303 nm excitation.





Figure S5. The Tauc plots for (TTA)_2_SbCl_5_ in the initial state and after decompression.





Figure S6. (a) The Tauc plots of indirect bandgap at 1 atm, and (b) the evolution of the indirect bandgap with pressure.





Figure S7. Rietveld refinements results under 1 atm, 0.5 GPa, 1.4 GPa and 2.4 GPa.





Figure S8. Rietveld refinements results under 4.6 GPa, 5.2 GPa, 6.8 GPa and 7.6 GPa.





Figure S9. Rietveld refinements results under 8.5 GPa, 9.7 GPa, 10.6 GPa and 16.1 GPa.





Figure S10. Evolution of the distance between different [SbCl_5_]^2-^ inorganic polyhedron with pressure.





Figure S11. Evolution of the distance between different [SbCl_5_]^2-^ inorganic polyhedron with pressure.


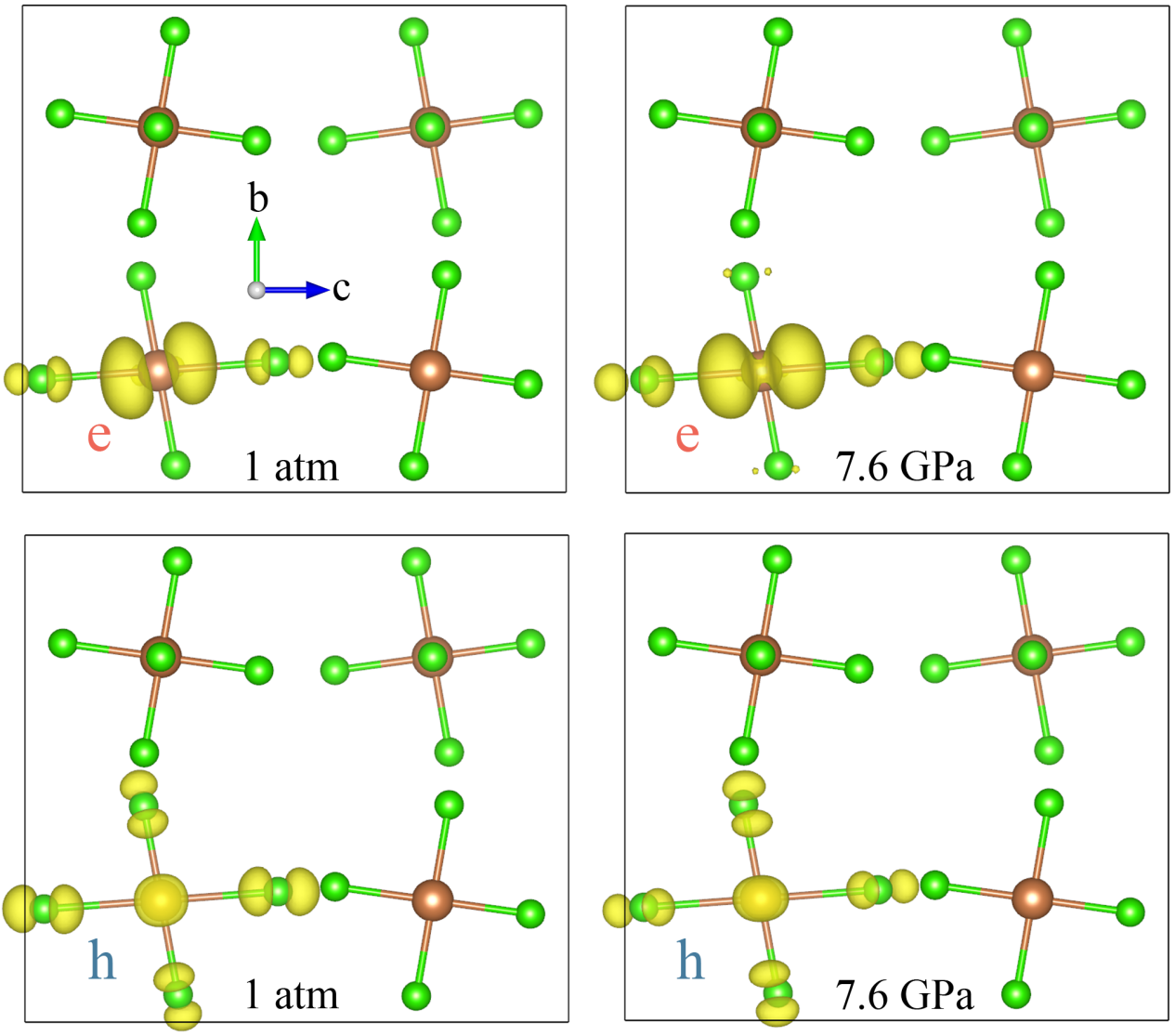


Figure S12. Wave function isosurface for electron and hole of (TTA)_2_SbCl_5_ in the excited state at 1 atm and 7.6 GPa.





Figure S13. Calculated spatial electron distributions of the HOMO (a); and the LUMO (b) of [SbCl_5_]^2-^.The red dashed coil contains the lone pair of electrons associated with Sb.


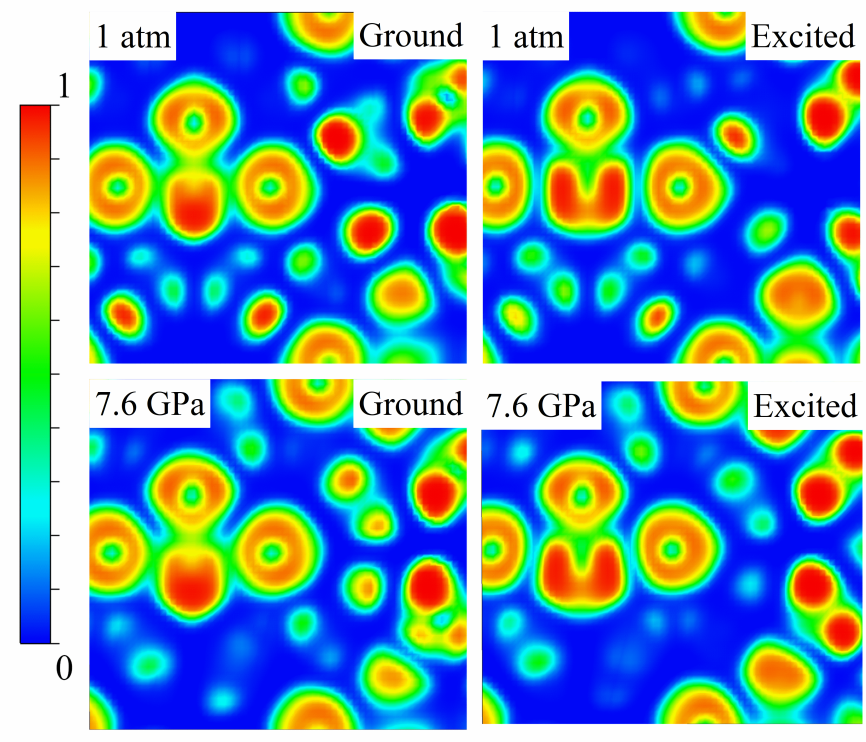


Figure S14. The electron localization function of the ground state and excited state structures of (TTA)_2_SbCl_5_ along the [011] direction at 1 atm and 7.6 GPa.

**References**

[1] Z. Li, Y. Li, P. Liang, T. Zhou, L. Wang, R.-J. Xie, *Chem. Mater.* **2019**, *31*, 9363-9371.

[2] a) G. Kresse, J. Furthmüller, *Comput. Mater. Sci.* **1996**, *6*, 15-50; b) G. Kresse, J. Furthmüller, *Phys. Rev. B* **1996**, *54*, 11169-11186.

[3] a) P. E. Blöchl, *Phys. Rev. B* **1994**, *50*, 17953-17979; b) J. P. Perdew, K. Burke, M. Ernzerhof, *Phys. Rev. Lett.* **1996**, *77*, 3865-3868.

[4] S. Grimme, *J. Comput. Chem.* **2006**, *27*, 1787-1799.

[5] a) M.-H. Du, *J. Chem. Phys.* **2019**, *151*; b) D. Yang, F. Wang, S. Li, Z. Shi, S. Li, *J. Phys. Chem. C* **2024**, *128*, 2223-2230.
